# Normative Approaches for Oral Health: Standards, Specifications, and Guidelines

**DOI:** 10.1177/00220345211049695

**Published:** 2021-10-25

**Authors:** G. Schmalz, N. Jakubovics, F. Schwendicke

**Affiliations:** 1 Department of Conservative Dentistry and Periodontology, University Hospital Regensburg, Regensburg, Germany; 2 Department of Periodontology, University of Bern, Bern, Switzerland; 3 School of Dental Sciences, Faculty of Medical Sciences, Newcastle University, Newcastle upon Tyne, UK; 4 Department of Oral Diagnostics, Digital Health and Health Services Research, Charité-Universitätsmedizin Berlin, Berlin, Germany

**Keywords:** COVID-19, deep learning/machine learning, materials science, outcomes research, clinical practice guidelines, artificial intelligence

## Abstract

Normative approaches have been developed with the aim of providing high-quality methods and strict criteria that, when applied correctly, lead to reliable results. Standards, specifications, and guidelines are needed to facilitate exchange of goods or information and secure comparability of data derived from different laboratories and sources. They are available along the whole flow from study development to test selection, study conduct, and reporting and are widely used for the evaluation of medical devices, market approval, and harmonization of terms and devices. Standards are developed by specific national and international organizations or by dedicated interest groups, mainly scientists in their respective fields. ISO (International Organization for Standardization) standards are developed following stringent regulations, and groups of experts formulate such standards. They should come from different areas (multistakeholder approach) to have as much and as broad input as possible and to avoid single-interest dominance. However, the presence of academia in such groups has been comparatively low. There is a clear need and responsibility of the oral health community to participate in the development of normative documents to provide methodological knowledge and experience, balance the interests of other stakeholders, and finally improve oral health. This will help to ensure that rapidly advancing fields of research, such as the oral health impacts of COVID-19 or the application of artificial intelligence in dentistry, benefit from standardization of approaches and reporting.

Discovery, development, and dissemination of knowledge in oral health must be based on stringent high-quality methodologies and strict and rigorous criteria. Normative approaches for scientific methods and criteria have been developed as one attempt to best meet such requirements and to ensure reliable results. Commonly used tools for such normative approaches are standards, specifications, or guidelines, which determine certain methods or approaches—usually along relevant definitions and criteria. They have a long tradition in oral health, and the role of using standards and their interrelation with the research field have recently been editorialized for dental materials science (Schmalz et al. 2021).

When funding and resources for research are stretched, it is critical that studies are well designed to ensure that the outcomes are robust and to avoid research waste (Glasziou et al. 2014). The importance of normative approaches has further become very clear in the COVID-19 pandemic, where they have been critical for quality control of protective equipment and risk assessment in dentistry and for the development of new diagnostic, therapeutic, and preventive strategies. The same is true for the new field of artificial intelligence (AI), where a lack of standards has led to inconsistencies in reporting that make it difficult to compare or replicate studies. This all has a decisive impact on the field of oral health.

Here, we discuss the use and need for normative approaches in oral health with the aim of raising awareness of the usefulness and value of these tools as well as their limitations. It is within the responsibility of the oral health research community to further develop and improve them.

## Standards and Quality Control

The term *standard* is used in many areas of daily life; it is often associated with (high) quality or to describe the presently accepted state of the art to ensure and control certain quality (standard model, standard of care, global standard, gold standard, ethical standards, etc.). The term is also used in the context of ensuring consistency (harmonized convention) for fixed values such as standard time, standard paper formats, or International Standard Book Number (ISBN). According to the *Encyclopedia Britannica*, the term *standard* covers different areas; for oral health, the following parts may apply the most: “something established by authority, custom, or general consent as a model or example” or as “a rule for the measure of quantity, weight, extent, value, or quality.” *Specification* means a “detailed precise presentation of something or of a plan or proposal for something.” Guidelines provide an “indication or outline of policy or conduct.” Normative approaches additionally comprise national or international legal regulations with a high degree of compulsoriness, such as medical device regulation in the European Union or the Food and Drug Administration (FDA) regulations for medical devices in the United States. These will not be covered here.

## Need for Normative Approaches

Normative approaches, such as standards, specifications, or guidelines, are meant to facilitate exchange of goods or information (nomenclature, terminology) and secure comparability of data derived from different laboratories and sources. They generally reflect the state of the art in their specific areas. For medical devices, they further set the quality level predominantly, but not exclusively, for safety and performance testing for established groups of market products.

Although the use of such documents is basically voluntary, standards can be and have been used to fulfill legal requirements for market access—for instance, to demonstrate compliance with essential requirements regarding the performance, safety, and quality of medical devices. In the context of the EU medical device regulation or related regulations (e.g., the US FDA), this is done within the framework of a subsidiarity approach. This means that responsibilities for defining specific tests and requirements are delegated to a level (here, standards organizations) at which better technical expertise is expected. This implies a partial delegation of responsibilities by society to bodies responsible for the development of standards. Altogether, standards, specifications, and guidelines are meant to contribute to a high level of oral health and to the quality and safety of dental care (Jones 2012) and are present all along the pipeline of research planning, conduct, and reporting, as well as the lifecycle of any medical device (Figure). Relevant standards for oral health care may, however, be important in fields such as sustainability and environmental protection. For instance, in the context of the Minamata Convention for reducing mercury in the environment, the phasedown of amalgam use is directly linked to a phase-in of amalgam separators, the quality of which is defined by an ISO standard (International Organization for Standardization; ISO 11143 2008).

**Figure.**
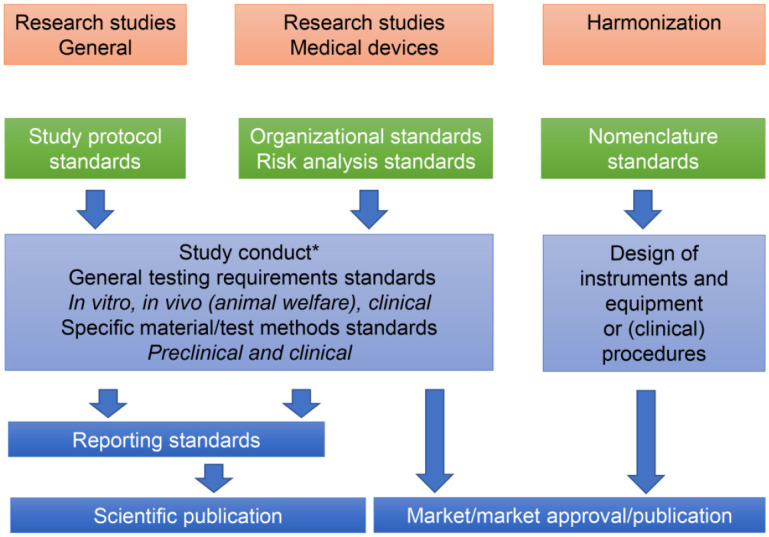
Standards are available along the whole flow from study development to test selection, study conduct, and reporting; they are generally used for medical device evaluation and market approval and for harmonization of terms and devices. These standards can be on a metalevel (e.g., general scientific or organizational standards and on terminology) or related to specific steps, devices, or clinical situations. Reporting of results can be done as a scientific publication or other press releases and publications (e.g., for harmonized clinical guidelines). Examples for single standards are listed in the Table. *Specific research topics may require methods that are not covered by standards, which lies in the responsibility of the scientist, but reporting standards should be followed.

A wide range of standards, specifications, and guidelines exists, and examples for the oral health field are listed in the Table. Guidelines for performing clinical treatments are not covered here. The relevant documents are itemized according to their specific areas, starting with the most general approach (PICO) used for the construction of the research question and for bibliographical search (da Costa Santos et al. 2007), followed by more specific standards for medical device testing, such as standards developed by ISO TC 106 (Technical Committee; dentistry) and ISO TC 194 (biological and clinical evaluation of medical devices), and finally covering data reporting and synthesis from various medical study types promoted by the EQUATOR Network (Enhancing the Quality and Transparency of Health Research). However, documents developed by other ISO TCs directly affect oral health care (e.g., in the context of COVID-19).

**Table.** Examples for Normative Approaches Relevant for Oral Health: Standards, Guidelines, or Specifications.

Area	Standard Designation and Acronym	Content and Title	Responsible Organization	Selected References
General approaches^a^	PICO strategy	Study design: P, patient or problem; I, intervention; C, control or comparison; O, outcome	DIG	da Costa Santos et al. (2007)
	ISO 14971 (2019)	Risk management medical devices	ISO	ISO 14971 (2019)
	ISO 13485 (2016)	Medical devices—quality management systems—requirements for regulatory purposes	ISO	ISO 13485 (2016)
	ISO 1942 (2020)	Terminology: dentistry—vocabulary	ISO	ISO 1942 (2020)
**Preclinical**				
General	OECD GLP	Principles of good laboratory practices and compliance monitoring	OECD	OECD (1999)
Technical	180 ISO standards	Laboratory test methods and requirements for medical devices, including materials, health care products, instruments, and dental equipment	ISO Technical Committee 106	ISO/TC 106 (2021)
Biological	ISO 10993 series (22 documents)	Biological and clinical evaluation of medical devices: basic principles and test methods	ISO Technical Committee 194	ISO/TC 194 (2021)
	OECD guidelines on health effects (20 documents)	OECD guidelines for testing chemical safety and biosafety	OECD	OECD (2021)
	ISO 7405 (2018)	Evaluation of biocompatibility of medical devices used in dentistry	ISO Technical Committee 106	ISO 7405 (2018)
	ADA/ANSI specification 41	Evaluation of biocompatibility of medical devices used in dentistry, see also ISO 7405	American Dental Association; American National Standards Institute	American Dental Association (2015)
In vivo regulations	ISO 10993-2 (2006)	Animal welfare	ISO Technical Committee 194	ISO 10993-2 (2006)
	DIRECTIVE 2010/63/EU	Protection of animals used for scientific purposes	European Union	European Commission (2019)
	NRC guide	The guide for the care and use of laboratory animals	US National Research Council	National Research Council (2011)
In vivo reporting	ARRIVE	Animals in research reporting in vivo experiments	DIG	Kilkenny et al. (2010)
**Clinical**				
General	GCP	Good clinical practice	ICH	ICH (2019)
Evaluating dental restorative materials	USPHS guidelines	US Public Health Service criteria for the clinical evaluation of dental restorative materials (1971)	US Public Health Service; US Department of Health, Education, and Welfare	Cvar and Ryge (2005)
	FDI criteria	FDI World Dental Federation: Recommendations for conducting controlled clinical studies of dental restorative materials	FDI World Dental Federation	Hickel et al. (2007)
Reporting	CONSORT	Consolidated Standards of Reporting Trials	DIG	Calvert et al. (2013); Schulz et al. (2010); Montgomery et al. (2018)
	STROBE	Strengthening the Reporting of Observational Studies in Epidemiology	DIG	von Elm et al. (2014)
	STARD (2015)	Standards for Reporting Diagnostic Accuracy Studies	DIG	EQUATOR Network (2021)
	TRIPOD	Transparent Reporting of a Multivariable Prediction Model for Individual Prognosis or Diagnosis	DIG	EQUATOR Network (2021)
Literature: review and meta-analysis	PRISMA	Preferred Reporting Items for Systematic Reviews and Meta-analyses	DIG	Page et al. (2021)

DIG, dedicated interest group; ICH, International Council for Harmonisation of Technical Requirements for Pharmaceuticals for Human Use; ISO, International Organization for Standardization; OECD, Organisation for Economic Cooperation and Development.aStudy protocol, organizational standards, and terminology.

## Developing Standards

Standards have been and are developed by specific national and international organizations, such as the ISO, IEC (International Electrotechnical Commission), or OECD (Organisation for Economic Cooperation and Development) on an international level, or by dedicated interest groups, mainly scientists of their respective fields. Here, we concentrate on ISO standards, but the conclusions are valid for equivalent national or international documents.

ISO standards are developed following stringent regulations (ISO 2021c). This distinguishes them from many other guidelines, and it is one reason why ISO (or similar) standards can be used to show compliance with national and international legislation. Standards relevant to oral health may be initiated by industry, dental practice, or the scientific community. Then, experts formulate such standards in working groups according to a defined schedule and format. Experts should come from different groups and areas (multistakeholder approach) to have as much and as broad input as possible and to avoid single-interest dominance. Developing standards or guidelines is mainly a consensus-based approach. The development workflow is based on the approval by national and regional standards organizations with a majority vote of 75% being needed.

## Pros and Cons

Standards represent the presently accepted (state of the art) quality level concerning performance of preclinical and clinical (product) testing (Schmalz et al. 2021) and provide methods and results for reporting of scientific studies, ensuring comparability and comprehensiveness and limiting reporting bias. Collections of standards can also be considered repositories of robust methods, “which are optimized for discriminatory power, reproducibility and comparability for use all over the world within constraints such as expense, time, equipment and expertise availability, yet still sufficient for purpose, ensuring a minimum confidence” (Schmalz et al. 2021).

In contrast, it is well understood that for many research projects methods that are not covered by standards must be used (Schmalz et al. 2021). Reporting should nevertheless be comprehensive and along reporting standards. Even if certain items cannot be fully reflected by the chosen study design, consideration should be given to them when reporting.

Furthermore, standards are models just like any other experimental method, always representing only a segment of reality. Therefore, predictability of the results from such, mainly laboratory, tests in terms of clinical relevance is a point of concern (Cesar, Della Bona, et al. 2017; Cesar, Hickel, et al. 2017). Within a scientific publication, the clinical relevance of any results obtained with a specific test always needs to be discussed conscientiously taking these limitations into account.

Many standards include fixed pass/fail criteria with the specified test method. Under the outlined limitations, this means a great responsibility achieving the right balance between aiming at a high quality level on the one hand and seeing the test limitations on the other. In the biological field, such fixed pass/fail criteria are less common; however, the test results are the basis for a clinical risk assessment, and in such cases data from successful market products may serve as benchmark.

A further problem is the time needed to develop a standard. The COVID-19 pandemic has highlighted the importance of being able to develop normative approaches quickly: for example, ISO/WD/TS^1^ 5798 (2021) is under development for the “Quality Practice for Detection of Severe Acute Respiratory Syndrome Coronavirus 2 (SARS-CoV-2) by Nucleic Acid Amplification Methods” and will include process steps for respiratory tract specimens. This will ultimately improve the level of confidence in COVID-19 testing.

## New Challenges

As mentioned, the development of normative approaches must respond quickly to newly emerging risks or new technologies. This is exemplified here by COVID-19 and AI, areas that are rapidly evolving.

COVID-19 was and is not only a challenge for developing strategies for the prevention and treatment of this disease; it also is a challenge for quality control of installed measures far beyond oral health care. Many national and international guidelines for the oral health care field have been issued in response to COVID-19 (e.g., by dental associations; Robertson et al. 2021). Risks of infection and transmission between persons and from/to health care personnel attracted the attention of normative organizations such as the American Society of Heating, Refrigerating and Air-Conditioning Engineers, the National Institute for Occupational Safety and Health, and the ISO. The last of which has made several standards relevant to COVID-19 freely available (ISO 2021b). Examples of relevant standards or regulations are compiled in the Appendix Table, from general requirements for public health, buildings ventilation, air quality, and room cleanliness to personal protection and dental equipment, such as high-flow/volume evacuation equipment. ISO 10637 (2018) specifies test methods and requirements for such equipment and distinguishes 3 classes based on the evacuation volume. There are indications that high-flow evacuation systems with around 300 L/min of air flow, such as class I of ISO 10637, significantly reduce spreading of droplet, splatter, and (potentially) aerosols into the dental environment (Graetz et al. 2021). Standards for using saliva as a diagnostic tool are developed under ISO/WD/TS 5798 (2021) and ISO/FDIS^2^ 4307 (2021).

In the case of AI, there is intense debate around standardization and regulation, mainly as current applications are not all robust, generalizable, and explainable; that is, they may suffer from undetected bias and performance gaps (Liu et al. 2020; Nagendran et al. 2020; Schwendicke, Chaurasia, et al. 2021). Generally, AI applications are approached in a similar way as other medical devices (e.g., non-AI software), with a risk-based approach considering patients, users, or third parties to ensure safety and performance (e.g., IEC 62304 2015, “Software Life Cycle”; ISO 14971, “Risk Management Medical Devices”). The ISO is presently working on 26 standards or technical reports around AI (ISO 2021a).

Besides risks, a value-based approach toward standardizing and regulating AI is taken in some areas of the world. The European Commission (2021) described a proposal for a regulation laying down harmonized rules on AI, with the specific objective “to set requirements specific to AI systems and obligations on all value chain participants to ensure that AI systems placed on the market and used are safe and respect existing law on fundamental rights and Union values.”

A range of other groups are active in increasing the robustness and applicability of medical AI via standards. The International Telecommunication Union with the World Health Organization installed an AI for Health focus group, which is working on standards, especially on benchmarking of medical AI. Benchmarking as outcomes-standardized testing will allow scrutinizing the generalizability and explainability of medical AI and help to critically appraise claimed performances. Standards on planning, conducting, and reporting AI studies have been consented by this group in joint efforts with IADR’s e-oral health network (Schwendicke, Singh, et al. 2021).

One specific difficulty arises when aiming to standardize dynamic AI (i.e., constantly learning AI). These systems are at risk such that retraining the AI may introduce unexpected errors or bias, varying performances, and catastrophic forgetting (Vokinger et al. 2021). In January 2021, the FDA issued an action plan for regulating dynamic AI, including the need to prespecify why and how retraining of AI is expected to improve safety and performance, as well as the planned methodology involved (algorithm change protocol). Moreover, standardized testing routines for dynamic AI and postapproval monitoring are suggested.

## Need to Get Involved

There is a clear need for the oral health scientific community to get involved to provide the necessary methodological knowledge and experience, to compensate for the interests of other stakeholders (e.g., economic interests), and to promote patients’ interests. Each working group within the ISO, for example, is open for experts from oral health research. However, attendance of participants from academia has been low versus that of manufacturers.

A possible reason for the low representation of academia is the lack of funding for the time devoted. Also, and possibly more important, developing standards is not sufficiently esteemed for its scientific reputation, partially because standards are published without naming authors and thus the output for the single scientist in terms of the scientific “currency” (publications) is limited.

Hence, and not only in this context, a new definition of “scientific impact” as a basis for reputation and for career development may be needed. Traditionally, scientific impact is based on publication metrics such as impact factors and *h*-factors. However, such metrics have well-documented deficiencies; therefore, scientific outputs other than research articles will grow in importance for assessing research effectiveness in the future (DORA 2012; Hicks et al. 2015). The Lund Declaration (European Commission 2015; Initiative for Science in Europe 2021) emphasized that research assessment practices should further value nonacademic impact to foster career progression. Valuable scientific input into the development of normative documents such as standards should be reflected appropriately, because here research output and scientific expertise directly improve the quality of oral health.

## Conclusions

Normative approaches, such as standards, specifications, and guidelines, play a major role in defining and guaranteeing the quality level in oral health care and research. National and international standards play a specific role in this context because they are developed under stringent conditions based on the consensus of different stakeholders and countries. Therefore, they can be used to show compliance with certain legal requirements. Standards and standard development have a long tradition but need to constantly evolve concomitantly. Despite the importance of such standards, the involvement of oral health academia is comparatively low, and better funding and recognition within the scientific community for this work are warranted. Oral health research should engage in standards setting and should build on standards, lifting the level of study conduct and reporting, which then increases research relevance and implementation.

## Author Contributions

G. Schmalz, N. Jakubovics, F. Schwendicke, contributed to conception, design, data acquisition and interpretation, drafted and critically revised manuscript. All authors gave final approval and agree to be accountable for all aspects of the work.

## Supplemental Material

sj-docx-1-jdr-10.1177_00220345211049695 – Supplemental material for Normative Approaches for Oral Health: Standards, Specifications, and Guidelines Click here for additional data file. Supplemental material, sj-docx-1-jdr-10.1177_00220345211049695 for Normative Approaches for Oral Health: Standards, Specifications, and Guidelines by G. Schmalz, N. Jakubovics and F. Schwendicke in Journal of Dental Research

## Notes

1. WD, working draft; TS, technical specification.

2. FDIS, final draft international standard.
