# Supplementary material for: Normative Approaches for Oral Health: Standards, Specifications, and Guidelines
Source: J Dent Res. 2021 Oct 25;101(5):489–94. doi: 10.1177/00220345211049695 (PMC9024015; doi:10.1177/00220345211049695)
Supplement: sj-docx-1-jdr-10.1177_00220345211049695 – Supplemental material for Normative Approaches for Oral Health: Standards, Specifications, and Guidelines [file sj-docx-1-jdr-10.1177_00220345211049695.docx]

Supplemental Appendix

**Normative approaches for oral health: Standards, specifications and guidelines**

Gottfried Schmalz, Nick Jakubovics, Falk Schwendicke

Appendix Table 1: Examples of standards relevant for COVID-19

| Area | Standard designation/  Acronym | Content/Title | Selected references |
| --- | --- | --- | --- |
| General requirements,  buildings ventilation, air quality | ISO/AWI* 5477 | Health information technology standards for Public Health Emergency Preparedness and Response | ISO/AWI 5477 2021 |
|  | ISO/WD** TR^§^ 5202 | Building resilience strategies related to public health emergencies | ISO/WD/TR 5202 2021 |
|  | ISO 22320 | Emergency management procedures are covered in a series of standards | ISO 22320 2018 |
|  | ASHRAE Guidelines and Standards | Ventilation, air conditioning, in-room air cleaners | ASHRAE 2021 |
| Personal protection | ISO/PAS^§§^ 45005 | Personal Protection Equipment in general to protect work-related health, safety and well-being | ISO 15005 2020 |
|  | ISO 13688 | General requirements for protecting clothing | ISO 13688 2013 |
|  | ISO 374-5 | Protective gloves against dangerous chemicals and micro-organisms | ISO 374-5 2016 |
|  | EN 149 FFP | Filtering Face Pieces, Respiratory protective devices – filtering half masks to protect against particles (“FFP masks”) | CEN-CENELEC 2009 |
|  | CWA^#^ 17553 | Community face coverings - Guide to minimum requirements, methods of testing and use | CEN-CENELEC 2020 |
|  | NIOSH FFP | Filtering Face Piece respirators | The National Institute for Occupational Safety and Health 2019 |
| Dental equipment | ISO 10637 | Dental suction units | ISO 10637 2018 |
| Diagnostics | ISO/WD TS^##^ 5798 | Quality Practice for detection of Severe Acute Respiratory Syndrome Coronavirus 2 (SARS-CoV-2) by nucleic acid amplification methods | ISO 5798 2021 |
|  | ISO/FDIS^###^ 4307 | Molecular in vitro diagnostic examinations — Specifications for pre-examination processes for saliva — Isolated human DNA | ISO 4307 2021 |

* AWI = Approved new work item

** WD = Working draft

^§^ TR = Technical Report, contains information, which may include data obtained from a survey, for example, or from an informative report, or information of the perceived “state of the art”.

^§§^ PAS = Publicly Available Specification

^#^ CWA = CEN Workshop Agreement

^##^ TS = Technical specification

^###^ FDIS = Final Draft International Standard

**Appendix References**

American Society of Heating, Refrigerating and Air-Conditioning Engineers. 2021. Covid-19: One page guidance documents. 180 Technology Parkway Peachtree Corners, GA 30092 [accessed June 2021]. https://www.ashrae.org/technical-resources/covid-19-one-page-guidance-documents.

CEN-CENELEC CWA 17553. 2020. Community face coverings - Guide to minimum requirements, methods of testing and use. Rue de la Science 23, B - 1040 Brussels, Belgium [accessed June 2021]. https://www.cencenelec.eu/covid19/Pages/default.aspx

CEN-CENELEC EN 149. 2009 Respiratory protective devices – filtering half masks to protect against particles - requirements, testing, marking (commonly referred to as ‘ffp masks’). Rue de la Science 23, B - 1040 Brussels, Belgium [accessed June 2021]. https://standards.cen.eu/dyn/www/f?p=204:110:0::::FSP_PROJECT:32928&cs=1B0AB06FEB70E43960D46D1198C37CC09.

ISO 374-5. 2016. Protective gloves against dangerous chemicals and micro-organisms — part 5: Terminology and performance requirements for micro-organisms risks. Geneva, Switzerland: ISO Central Secretariat. [accessed June 2021]. https://www.iso.org/obp/ui#iso:std:iso:374:-5:ed-1:v1:en.

ISO 10637. 2018. Dentistry — central suction source equipment. Geneva, Switzerland: ISO Central Secretariat. [accessed June 2021]. https://www.iso.org/standard/68461.html.

ISO 13688. 2013. Protective clothing — general requirements. Geneva, Switzerland: ISO Central Secretariat. [accessed June 2021]. https://www.iso.org/obp/ui#iso:std:iso:13688:ed-2:v1:en.

ISO 22320. 2018. Security and resilience — emergency management — guidelines for incident management. Geneva, Switzerland: ISO Central Secretariat. [accessed June 2021]. https://www.iso.org/obp/ui#iso:std:iso:22320:ed-2:v1:en.

ISO/AWI 5477. 2021. Health informatics — reference standards portfolio(rsp)-public health emergency preparedness and response information system — rsp-ph epr information system. Geneva, Switzerland: ISO Central Secretariat. [accessed June 2021]. https://www.iso.org/standard/81303.html.

ISO/FDIS 4307. 2021. Molecular in vitro diagnostic examinations — specifications for pre-examination processes for saliva — isolated human DNA. Geneva, Switzerland: ISO Central Secretariat. [accessed June 2021]. https://www.iso.org/standard/79865.html.

ISO/PAS 45005. 2020. Occupational health and safety management — general guidelines for safe working during the covid-19 pandemic. Geneva, Switzerland: ISO Central Secretariat. [accessed June 2021]. https://www.iso.org/standard/64286.html.

ISO/WD/TR 5202. 2021. Buildings and civil engineering works — building resilience strategies related to public health emergencies — compilation of relevant information. Geneva, Switzerland: ISO Central Secretariat. [accessed June 2021]. https://www.iso.org/standard/80990.html.

ISO/WD/TS 5798. 2021. Quality practice for detection of severe acute respiratory syndrome coronavirus 2 (sars-cov-2) by nucleic acid amplification methods. Geneva, Switzerland: ISO Central Secretariat. [accessed June 2021]. https://www.iso.org/standard/81712.html.

The National Institute for Occupational Safety and Health. 2019. Filtering face piece respirators. [accessed]. https://www.cdc.gov/niosh/npptl/topics/respirators/disp_part/default.html.
